# Supplementary material for: Baveno Criteria Spare Endoscopies Among Patients With Compensated Cirrhosis Within a Large US Healthcare System
Source: Gastro Hep Adv. 2026 Apr 1;5(7):100945. doi: 10.1016/j.gastha.2026.100945 (PMC13213294; doi:10.1016/j.gastha.2026.100945)
Supplement: Supplementary Table [file mmc1.pdf]

Supplementary Tables:

Table S1: Spared endoscopies when applying Baveno VI criteria

	**Spared EGD, n (%)**	**Missed varices needing treatment, n (%)**
Baveno VI	90 (21.0)	1 (1.1)
