# Supplementary material for: Baveno Criteria Spare Endoscopies Among Patients With Compensated Cirrhosis Within a Large US Healthcare System
Source: Gastro Hep Adv. 2026 Apr 1;5(7):100945. doi: 10.1016/j.gastha.2026.100945 (PMC13213294; doi:10.1016/j.gastha.2026.100945)
Supplement: Extended PDF [file mmc2.pdf]

Gastro Hep Advances 2026;5:100945

ORIGINAL RESEARCH—CLINICAL

# Baveno Criteria Spare Endoscopies Among Patients With Compensated Cirrhosis Within a Large US Healthcare System

Marcia Leung,[1,2] Mehrnaz Siavoshi,[3] Nathan Billet,[1] Xiaoran Li,[1] Elijah Wade,[3] Sreepriya Balasubramanian,[4] Varun Saxena,[1] Suk Seo,[4] Krisna Chai,[4] Brock MacDonald,[4] Jeffrey Lee,[1,§] and Nizar Mukhtar[1,§]

*[1]Department of Gastroenterology, Kaiser Permanente San Francisco, San Francisco, California; [2]The Queen's Medical Center, Honolulu, Hawaii; [3]Division of Research, Kaiser Permanente Northern California, Oakland, California; and [4]Department of Gastroenterology, Kaiser Permanente Northern California, Roseville, California*

**BACKGROUND AND AIMS:** The Baveno criteria have been validated in populations outside of the United States and have been shown to accurately rule out clinically significant portal hypertension (CSPH) and high-risk varices. The criteria have been adopted by European and US guidelines. We aim to evaluate the performance characteristics of the Baveno VI criteria in a large US healthcare system. **METHODS:** Patients evaluated within Kaiser Permanente Northern California with cirrhosis (liver stiffness measurement ≥10 kPa) who underwent a FibroScan between 2016 and 2022 and an upper endoscopy within 12 months were included. Endoscopic and imaging findings were assessed. We evaluated the performance characteristics of the Baveno VI criteria for ruling out CSPH based on endoscopic and radiologic evidence consistent with CSPH. **RESULTS:** Among 428 patients, the sensitivity of the Baveno VI criteria for ruling out CSPH was determined to be 84.8%. The sensitivity for identifying medium/large varices was 95.1%. Applying Baveno criteria would spare 90 endoscopies. **CONCLUSION:** Application of the Baveno criteria spares 21% of unnecessary endoscopies with a low risk of missing high-risk varices, confirming that the Baveno criteria can be confidently adopted in the United States to rule out CSPH.

*Keywords:* Clinically Significant Portal Hypertension; Varices; Carvedilol; Transient Elastography; FibroScan

## Introduction

The consequences of portal hypertension, especially variceal bleeding, are a major cause of morbidity and mortality in patients with cirrhosis. Prior to 2015, US gastroenterology practice guidelines have recommended screening all patients with cirrhosis for varices through upper endoscopy to identify those who warrant prophylactic measures to reduce the risk of variceal bleeding (eg, esophageal band ligation and/or initiation of nonselective beta-blockers). However, the prevalence of high-risk varices that require treatment in patients with compensated advanced chronic liver disease (cACLD) is low, with a yield of 10% on upper endoscopy.[1]

Thus, based on a series of consensus meetings, the Baveno VI criteria were established to noninvasively identify patients with cACLD who may have clinically significant portal hypertension (CSPH) ruled out, ultimately sparing unnecessary endoscopies in patients deemed low risk for high-risk varices.[2] Prior studies have shown a correlation between liver stiffness as measured by transient elastography and hepatic venous pressure gradient (HVPG), with liver stiffness ≥20 kPa being predictive of CSPH with a positive predictive value (PPV) of 93%.[3] The Baveno VI consensus states that liver stiffness measured by vibration-controlled transient elastography (VCTE) along with platelet count may sufficiently and accurately rule out CSPH. Thus, in 2024, the American Association for the Study of Liver Diseases updated their guidelines to reflect these recommendations.[2,4]

Although current US guidelines reflect the Baveno recommendations, which have reliably identified patients with high-risk varices in European and Asian populations,[5] there have been no large validation studies of the Baveno criteria in a diverse and representative US population.[6–8,9] Validation of these criteria is especially important given the potential to reduce patient harm from unnecessary procedures and the associated healthcare burden.

The aim of this cross-sectional study is to determine the sensitivity of the Baveno VI criteria (ie, platelet count $>150 \times 10^9$/L and liver stiffness measurement [LSM] $<20$ kPa) for ruling out CSPH in a large, diverse cohort of patients with cirrhosis in the United States.[10]

[§]Denotes co-senior authors.

***Abbreviations used in this paper:*** **cACLD, compensated advanced chronic liver disease; CSPH, clinically significant portal hypertension; HVPG, hepatic venous pressure gradient; LSM, liver stiffness measurement; NPV, negative predictive value; PPV, positive predictive value; VCTE, vibration-controlled transient elastography.**

**Most current article**

**© 2026 The Authors. Published by Elsevier Inc. on behalf of American Gastroenterological Association Institute. This is an open access article under the CC BY license (http://creativecommons.org/licenses/by/4.0/).**
**2772-5723**
https://doi.org/10.1016/j.gastha.2026.100945

## Methods

This retrospective cross-sectional study was conducted at Kaiser Permanente Northern California. This study (IRBNet ID 2102596-5) was reviewed by the Kaiser Permanente Institutional Review Board (IRB# 00001045) and determined to be exempt from full review under 45 CFR §46.104(d)(4) (secondary research involving identifiable private information). The requirement for informed consent was waived due to the retrospective design and minimal risk to participants. All data were accessed and analyzed in accordance with institutional privacy standards, and patient confidentiality was maintained throughout the study. We included adult patients, 18 years or older, who underwent VCTE (FibroScan) between January 1, 2016, and December 30, 2022, and had a LSM of ≥10 kPa. All patients were also secondarily chart reviewed to ensure that the diagnosis of cirrhosis was accurate based on review of available liver biopsy reports, calculated FIB-4 score, available imaging for liver nodularity, and imaging reports for signs of portal hypertension. Patients met criteria for cirrhosis if they had a liver biopsy showing cirrhosis any time preceding VCTE or by meeting 2 of the 3 criteria: (1) LSM ≥10 on FibroScan, (2) liver surface nodularity or signs of collateralization on imaging within 1 year of VCTE (shunt, umbilical vein recanalization, rectal, or gastroesophageal varices), or (3) FIB-4 score >3.25 based on labs closest to FibroScan date.

All patients included also had an esophagogastroduodenoscopy and complete blood count within 12 months of the FibroScan date. A minimum follow-up period of 6 months after the FibroScan date was required for inclusion. Exclusion criteria included prior diagnosis of decompensated liver disease (defined as Child-Pugh B or C, or evidence of ascites, encephalopathy, or prior variceal hemorrhage), noncirrhotic portal hypertension, hepatocellular carcinoma, mesenteric or splenic vein thromboses, liver transplant, history of transjugular intrahepatic portosystemic shunt, and symptomatic alcohol-associated hepatitis.

Patient data, including demographic characteristics, clinical details, and imaging and endoscopy findings, were extracted from electronic medical records. The presence and size of gastroesophageal varices were determined from documented impressions in esophagogastroduodenoscopy reports. Varices were deemed small if they were straight, collapsed with air insufflation, and occupied less than one-third of the esophageal lumen. Medium varices were defined as enlarged tortuous varices that occupied less than one-third of the esophageal lumen. Large varices were defined as enlarged coil-shaped varices that occupied more than one-third of the esophageal lumen. Furthermore, there were no patients with small varices with red wale signs, which are considered high-risk varices.

The primary outcomes were the sensitivity, specificity, PPV, and negative predictive value (NPV) of the Baveno VI (platelet count $>150 \times 10^9$/L and LSM <20 kPa) criteria for ruling out CSPH, defined by endoscopic or radiological findings. Endoscopic findings consistent with CSPH include gastroesophageal varices and/or portal hypertensive gastropathy. Radiological evidence of CSPH includes portosystemic shunts or splenomegaly.

Descriptive statistics (counts, means, standard deviations, medians, and interquartile ranges) were calculated to compare demographic and clinical differences among patients who did or did not meet Baveno VI criteria. Kruskal-Wallis and chi-square *P* values were calculated to assess differences between patients who met or did not meet criteria. Sensitivity, specificity, PPV, and NPV, along with their 95% confidence intervals, were calculated for each set of criteria. Statistical significance was defined as a 2-sided *P* value of <.05 for all statistical comparisons. All analyses were performed using SAS version 9.4.

## Results

A total of 428 patients with the diagnosis of cirrhosis who met inclusion criteria were included in the final study cohort. The prevalence of CSPH, defined as either endoscopic evidence of gastroesophageal varices or portal hypertensive gastropathy or radiologic evidence of portosystemic shunts or splenomegaly, was 66.1% in all patients. The mean age was 62.53 years (±9.6); 49.1% were female, and 24.5% were Hispanic. The mean LSM was 25.38 (±14.4) kPa (Table 1).The most common etiologies of cirrhosis were viral hepatitis (41.8%), metabolic-associated steatohepatitis (34.1%), and alcohol-related liver disease (15.0%). Additionally, 85.4% of the cohort was classified as overweight or obese (Table 1).

Among those with presumptive CSPH by Baveno VI criteria, there was a larger proportion of patients with alcohol-related liver disease, a smaller proportion of patients with “other” as etiology for liver disease, which includes autoimmune and metabolic liver disease (eg, alpha-1 antitrypsin deficiency, Wilson disease) and unknown (Table 1). Patients in whom CSPH was ruled out based on Baveno VI criteria were more likely to be older and more likely to be diabetic, but there were no differences noted in regard to sex, race/ethnicity, body mass index, or current alcohol use compared to those who did not. Patients who did not meet Baveno VI criteria for excluding CSPH had a significantly higher prevalence of portal hypertensive gastropathy, esophageal varices, application of band ligation, and evidence of portal hypertension on imaging (Table 2). There was no significant difference in the prevalence of gastric varices when comparing patients who met Baveno VI criteria and those who did not (Table 2).

Applying the Baveno VI criteria to this cohort would spare 21.0% of endoscopies. Applying the Baveno VI criteria led to a 1.1% missed detection of medium/large varices, suggesting these criteria can effectively help identify low-risk individuals who may forgo endoscopy (Supplementary Table). The sensitivity of the Baveno VI criteria for ruling out CSPH based on endoscopic or imaging findings was 84.8% (Table 3). Of those who had CSPH ruled out based on application of the Baveno VI criteria, 47.8% still had endoscopic or imaging evidence of CSPH. In the majority of cases, this was evidenced by the presence of portal hypertensive gastropathy, collaterals on imaging, or small esophageal varices. Because of this, the NPV of the Baveno VI criteria was low at 52.2%. However, when evaluating the Baveno VI criteria for ruling out medium-large varices, the sensitivity was 95.1%, and the NPV was 97.9% (Table 4).

**Table 1.** Baseline Characteristics

| | All patients N = 428 | Baveno VI criteria met N = 90 | Baveno VI criteria not met N = 338 | *P* value |
|---|---|---|---|---|
| Age at FibroScan (y), mean (SD) | 62.53 (9.56) | 65.63 (10.15) | 61.71 (9.24) | <.001[a] |
| Sex (female), n (%) | 210 (49.1) | 48 (53.3) | 162 (47.9) | .362[b] |
| Race/ethnicity[c], n (%) | | | | .634[b] |
| Asian | 58 (13.6) | 13 (14.4) | 45 (13.3) | |
| Black | 22 (5.1) | 7 (7.8) | 15 (4.4) | |
| Hispanic | 105 (24.5) | 21 (23.3) | 84 (24.9) | |
| White | 220 (51.4) | 46 (51.1) | 174 (51.5) | |
| Other | 23 (5.4) | 3 (3.3) | 20 (5.9) | |
| Etiology of liver disease, n (%) | | | | .011[b] |
| Alcohol | 64 (15.0) | 6 (6.7) | 58 (17.2) | |
| MASH | 146 (34.1) | 30 (33.3) | 116 (34.3) | |
| Viral hepatitis | 179 (41.8) | 40 (44.4) | 139 (41.1) | |
| Other | 36 (8.4) | 14 (15.6) | 22 (6.5) | |
| Unknown | 3 (0.7) | 0 (0.0) | 3 (0.9) | |
| BMI | | | | .432[a] |
| Mean (SD) | 30.91 (6.00) | 31.53 (6.23) | 30.74 (5.93) | |
| Underweight <18.5 kg/m$^2$, n (%) | 4 (1.0) | 1 (1.1) | 3 (0.9) | |
| Normal 18.5–24.9 kg/m$^2$, n (%) | 56 (13.7) | 8 (9.0) | 48 (15.0) | |
| Overweight 25.0–29.9 kg/m$^2$, n (%) | 143 (34.9) | 37 (41.6) | 106 (33.0) | |
| Obese ≥30.0 kg/m$^2$, n (%) | 207 (50.5) | 43 (48.3) | 164 (51.1) | |
| Missing | 18 | 1 | 17 | |
| Diabetes, n (%) | 189 (44.2) | 45 (50.0) | 144 (42.6) | .209[b] |
| Alcohol use, yes, n (%) | 120 (28.6) | 24 (27.0) | 96 (29.1) | .341[b] |
| Platelet count 10$^9$/L, mean (SD) | 145.40 (65.50) | 212.80 (62.98) | 127.46 (53.40) | <.001[a] |
| FIB-4 score, mean (SD) | 4.05 (2.55) | 2.23 (0.96) | 4.53 (2.62) | <.001[a] |
| MELD-NA, mean (SD) | 8.71 (2.86) | 8.21 (2.52) | 8.85 (2.93) | .027[a] |
| MELD-3.0, mean (SD) | 9.14 (2.83) | 8.64 (2.32) | 9.28 (2.94) | .043[a] |
| Creatinine, mean (SD) | 0.90 (0.58) | 0.89 (0.23) | 0.90 (0.64) | .022[a] |
| Total bilirubin, mean (SD) | 0.92 (0.47) | 0.77 (0.38) | 0.96 (0.48) | <.001[a] |
| INR, mean (SD) | 1.12 (0.15) | 1.08 (0.17) | 1.13 (0.14) | <.001[a] |
| Sodium, mean (SD) | 139.59 (2.98) | 139.13 (3.21) | 139.72 (2.91) | .117[a] |
| Albumin, mean (SD) | 3.92 (0.44) | 4.05 (0.38) | 3.89 (0.44) | .011[a] |
| Liver stiffness (kPa), mean (SD) | 25.38 (14.37) | 14.79 (2.73) | 28.20 (14.89) | <.001[a] |
| Esophageal varices, n (%) | 146 (34.1) | 15 (16.7) | 131 (38.8) | <.001[b] |
| Medium/large, n (%) | 41 (9.6) | 2 (2.2) | 39 (11.5) | <.001[b] |
| Any varices, n (%) | 150 (35.0) | 15 (16.7) | 135 (39.9) | <.001[b] |

BMI, body mass index; FIB-4, fibrosis-4; INR, international normalized ratio; MASH, metabolic dysfunction-associated steatohepatitis; MELD-Na, model for end-stage liver disease-sodium; SD, standard deviation.
[a]Kruskal-Wallis *P* value.
[b]Chi-square *P* value.
[c]Asian includes Native Hawaiian and Pacific Islander. Other includes American Indian, Alaska Native, and multiracial.

**Table 2.** Upper Endoscopy or Radiological Findings Suggestive of Clinically Significant Portal Hypertension

| | | Baveno VI | | |
|---|---|---|---|---|
| n (%) | All patients N = 428 | Criteria met N = 90 | Criteria not met N = 338 | *P* value[a] |
| Portal hypertensive gastropathy | 164 (38.3) | 24 (26.7) | 140 (41.4) | .011 |
| Varices | | | | |
| Any varices | 150 (35.1) | 15 (16.7) | 135 (39.9) | <.001 |
| Esophageal varices | 146 (34.1) | 15 (16.0037) | 131 (38.8) | <.001 |
| Gastric varices | 12 (2.8) | 0 (0) | 12 (3.2) | .201 |
| Esophageal varices size | | | | <.001 |
| Small | 105 (24.5) | 13 (14.4) | 92 (27.2) | |
| Medium/large | 41 (9.6) | 2 (2.2) | 39 (11.5) | |
| Variceal band ligation applied | 21 (4.9) | 1 (1.1) | 20 (5.9) | .061 |
| Collaterals on imaging (shunt, umbilical vein recanalization, rectal, or gastroesophageal varices) | 136 (31.8) | 18 (20.0) | 220 (65.1) | .007 |
| CSPH as indicated by any of the above criteria | 283 (66.1) | 43 (47.8) | 240 (71.0) | <.001 |

[a]Chi-square *P* value.

**Table 3.** Sensitivity, Specificity, PPV, and NPV for Predicting Clinically Significant Portal Hypertension Using Baveno VI Criteria

| | Sensitivity (95% CI) | Specificity (95% CI) | PPV (95% CI) | NPV (95% CI) |
|---|---|---|---|---|
| Baveno VI | 84.8 [80.1, 88.8] | 32.4 [24.9, 40.7] | 71.0 [68.4, 73.5] | 52.2 [43.2, 61.1] |

CI, confidence interval.

## Discussion

Our study is the first to validate the Baveno VI criteria in a large, diverse US cohort. In regard to our primary endpoints of performance characteristics of the Baveno VI criteria, we have confirmed the high sensitivity of the Baveno VI criteria for ruling out CSPH and its high NPV for ruling out medium-large varices.

Since the institution of the Baveno VI criteria, there have been several published manuscripts testing its performance, and all have shown a high NPV between 93% and 100% for excluding high-risk varices.[9,11] We have also similarly found a high NPV of 97.9% for the Baveno VI criteria for excluding medium/large varices. If implemented, the Baveno VI criteria can lead to considerable cost savings over a patient's lifetime by sparing unnecessary surveillance endoscopies, which vary in cost from $1000 to $4000 in the United States depending on setting and insurance coverage. Sparing unnecessary endoscopies by using the Baveno criteria also contributes to efforts of reducing the carbon footprint of endoscopy procedures, as each gastroenterology procedure is reported to generate approximately 1.5 kg of plastic waste.[12]

Our findings confirm that the vast majority of surveillance endoscopies being performed on patients with cACLD are unnecessary. Of all patients who underwent endoscopy, only 9.6% had varices that were eligible for treatment with band ligation, suggesting that about 90% of all upper endoscopies performed on patients with cACLD may potentially be spared.

Prior studies have evaluated different thresholds for platelets and LSM, or other adjunctive measures such as spleen stiffness to exclude more patients from unnecessary endoscopies. They have proposed 5% as the acceptable rate of missed varices needing treatment (ie, medium or large varices). Our study has confirmed a rate of missed varices needing treatment that is well below this value at 1.1%, suggesting that the criteria may be liberalized to spare more unnecessary endoscopies, while still remaining below the 5% acceptable rate of missed varices needing treatment.

We found a prevalence of medium/large varices (eg, varices needing treatment) of 9.6%, which is slightly lower than the 12.0% reported on prior studies performed outside of the United States.[13] Overall, we also had a lower rate of varices at 35% compared to the average of 41.5% in prior studies.

We found that the application of the Baveno VI criteria in our population would potentially save 21.0% of endoscopies, which is slightly less than the 31.3% spared by the criteria in previously published studies.[6,14]

The strengths of this study include a large cohort of patients in an integrated hospital system with a diverse patient population, who in addition to having a LSM cutoff of $\geq 10$ on VCTE as a definition of cirrhosis, also had the diagnosis of cirrhosis verified through review of patient records. When more than one etiology of liver disease was evident, the dominant etiology was decided upon through review of the chart by at least 2 physicians. The limitations of the study include retrospective nature subjecting to bias, reliance on the availability of endoscopic and imaging findings, and LSM measurements, and the absence of available HVPG measurements to confirm the noninvasive diagnosis of CSPH. Case reports suggest that portosystemic shunts and varices can persist despite etiology removal and normalization of the HVPG, suggesting that we may potentially be overestimating the prevalence of CSPH if using endoscopic and imaging criteria.[15] However, absence of HVPG reflects US community practice where HVPG measurement is not readily accessible. Furthermore, this study was performed in an integrated health system in the Northern California region with standardized care pathways, thus findings may not be generalizable to nonintegrated or fee-for-service systems.

## Conclusion

Our findings confirm that the Baveno VI criteria have high sensitivity for ruling out CSPH and high-risk varices, offering a reliable method to identify low-risk patients who can safely avoid unnecessary endoscopies. Future areas of research include determining if LSM or noninvasive studies can predict future decompensation or mortality, as well as

**Table 4.** Sensitivity, Specificity, PPV, and NPV for Predicting Medium/Large Esophageal Varices

| | Sensitivity (95% CI) | Specificity (95% CI) | PPV (95% CI) | NPV (95% CI) |
|---|---|---|---|---|
| Baveno VI | 95.1 [83.5, 99.4] | 22.7 [18.7, 27.2] | 11.5 [10.7, 12.5] | 97.9 [91.8, 99.4] |

CI, confidence interval.

refining these criteria within specific subgroups to improve their specificity.

## Supplementary Materials

Material associated with this article can be found, in the online version, at https://doi.org/10.1016/j.gastha.2026.100945.

## References

1. Jakab SS, Garcia-Tsao G. Screening and surveillance of varices in patients with cirrhosis. Clin Gastroenterol Hepatol 2019;17(1):26–29.
2. De Franchis R, Bosch J, Garcia-Tsao G, et al. Baveno VII – renewing consensus in portal hypertension. J Hepatol 2022;76(4):959–974.
3. Kumar A, Khan NM, Anikhindi SA, et al. Correlation of transient elastography with hepatic venous pressure gradient in patients with cirrhotic portal hypertension: a study of 326 patients from India. World J Gastroenterol 2017;23(4):687–696.
4. Kaplan DE, Ripoll C, Thiele M, et al. AASLD Practice Guidance on risk stratification and management of portal hypertension and varices in cirrhosis. Hepatology 2024;79(5):1180–1211.
5. Villanueva C, Albillos A, Genescà J, et al. $\beta$ blockers to prevent decompensation of cirrhosis in patients with clinically significant portal hypertension (PREDESCI): a randomised, double-blind, placebo-controlled, multicentre trial. Lancet 2019;393(10181):1597–1608.
6. Gaete MI, Díaz LA, Arenas A, et al. Baveno VI and Expanded Baveno VI criteria successfully predicts the absence of high-risk gastro-oesophageal varices in a Chilean cohort. Liver Int 2020;40(6):1427–1434.
7. Szakács Z, Erőss B, Soós A, et al. Baveno criteria safely identify patients with compensated advanced chronic liver disease who can avoid variceal screening endoscopy: a diagnostic test accuracy meta-analysis. Front Physiol 2019;10:1028.
8. Vutien P, Barnard Giustini A, Kim NJ, et al. Validation and expansion of Baveno VII criteria for cACLD and CSPH based on liver stiffness and platelet count: correlation with risk of hepatic decompensation and death. Hepatology 2025;82:422–437.
9. Jangouk P, Turco L, De Oliveira A, et al. Validating, deconstructing and refining Baveno criteria for ruling out high-risk varices in patients with compensated cirrhosis. Liver Int 2017;37(8):1177–1183.
10. Huang DQ, Terrault NA, Tacke F, et al. Global epidemiology of cirrhosis — aetiology, trends and predictions. Nat Rev Gastroenterol Hepatol 2023;20(6):388–398.
11. Bai W, Abraldes JG. Noninvasive assessment oesophageal varices: impact of the Baveno VI criteria. Curr Opin Gastroenterol 2022;38(3):206–215.
12. Gayam S. Environmental impact of endoscopy: “scope” of the problem. Am J Gastroenterol 2020;115(12):1931–1932.
13. Augustin S, Pons M, Genesca J. Validating the Baveno VI recommendations for screening varices. J Hepatol 2017;66(2):459–460.
14. Augustin S, Pons M, Maurice JB, et al. Expanding the Baveno VI criteria for the screening of varices in patients with compensated advanced chronic liver disease. Hepatology 2017;66(6):1980–1988.
15. Olivas P, Soler-Perromat A, Tellez L, et al. Persistent varices in cured patients: understanding the role of hepatic venous pressure gradient. JHEP Rep 2024;6(10):101170.

**Received May 9, 2025. Accepted March 23, 2026.**

**Correspondence:**
Address correspondence to: Nizar Mukhtar, MD, Department of Gastroenterology, Kaiser Permanente San Francisco, 2350 Geary Boulevard, 2nd Floor, San Francisco, California 94115. e-mail: nizar.a.mukhtar@kp.org.

**Conflicts of Interest:**
The authors disclose no conflicts.

**Funding:**
This study was funded by the Kaiser Permanente Northern California Graduate Medical Education Biostatistical Consulting Unit (NCI R37CA276306).

**Ethical Statement:**
The study was approved by the KPNC Institutional Review Board, with informed consent waived.

**Data Transparency Statement:**
Data, analytic methods, and study materials will be made available to other researchers upon request.

**Reporting Guidelines:**
The authors followed the Strengthening the Reporting of Observational Studies in Epidemiology (STROBE) reporting guidelines for cross-sectional studies to ensure accurate and transparent reporting of this research.

Gastro Hep Advances, Volume 5

## Supplemental information

# Baveno Criteria Spare Endoscopies Among Patients With Compensated Cirrhosis Within a Large US Healthcare System

**Marcia Leung, Mehrnaz Siavoshi, Nathan Billet, Xiaoran Li, Elijah Wade, Sreepriya Balasubramanian, Varun Saxena, Suk Seo, Krisna Chai, Brock MacDonald, Jeffrey Lee, and Nizar Mukhtar**

**Supplementary Tables:**

**Table S1: Spared endoscopies when applying Baveno VI criteria**

| | **Spared EGD, n (%)** | **Missed varices needing treatment, n (%)** |
|---|---|---|
| Baveno VI | 90 (21.0) | 1 (1.1) |

**Supplementary Tables:**

**Table S1: Spared endoscopies when applying Baveno VI criteria**

| | **Spared EGD, n (%)** | **Missed varices needing treatment, n (%)** |
|---|---|---|
| Baveno VI | 90 (21.0) | 1 (1.1) |
